# PTP1B triggers integrin-mediated repression of myosin activity and modulates cell contractility

**DOI:** 10.1242/bio.015883

**Published:** 2015-12-23

**Authors:** Ana E. González Wusener, Ángela González, Fumihiko Nakamura, Carlos O. Arregui

**Affiliations:** 1 IIB-INTECH, Universidad Nacional de San Martín, 1650 San Martín, Buenos Aires, Argentina; 2 Hematology Division, Department of Medicine, Brigham and Women's Hospital, Harvard Medical School, Boston, MA 02445, USA

**Keywords:** PTP1B, Src, FAK, Integrin, Myosin, Contractility

## Abstract

Cell contractility and migration by integrins depends on precise regulation of protein tyrosine kinase and Rho-family GTPase activities in specific spatiotemporal patterns. Here we show that protein tyrosine phosphatase PTP1B cooperates with β3 integrin to activate the Src/FAK signalling pathway which represses RhoA-myosin-dependent contractility. Using PTP1B null (KO) cells and PTP1B reconstituted (WT) cells, we determined that some early steps following cell adhesion to fibronectin and vitronectin occurred robustly in WT cells, including aggregation of β3 integrins and adaptor proteins, and activation of Src/FAK-dependent signalling at small puncta in a lamellipodium. However, these events were significantly impaired in KO cells. We established that cytoskeletal strain and cell contractility was highly enhanced at the periphery of KO cells compared to WT cells. Inhibition of the Src/FAK signalling pathway or expression of constitutive active RhoA in WT cells induced a KO cell phenotype. Conversely, expression of constitutive active Src or myosin inhibition in KO cells restored the WT phenotype. We propose that this novel function of PTP1B stimulates permissive conditions for adhesion and lamellipodium assembly at the protruding edge during cell spreading and migration.

## Abstract (precis)

**Summary:** Here we show that protein tyrosine phosphatase PTP1B cooperates with β3 integrin to transiently repress RhoA-myosin-dependent contractility, stimulating adhesion and lamellipodium assembly during cell spreading and migration.

## INTRODUCTION

Protein tyrosine phosphatases, including PTP1B, have been established as important regulators of integrin-mediated signal transduction implied in cytoskeletal rearrangements and cell migration (Larsen et al., 2003; Burridge et al., 2006; Arregui et al., 2013).

PTP1B is an endoplasmic reticulum (ER)-anchored enzyme whose access to substrates is partly dependent on the ER distribution and dynamics (Frangioni et al., 1992; Hernández et al., 2006; Anderie et al., 2007; Fuentes and Arregui, 2009; Nievergall et al., 2010; Haj et al., 2012; Monteleone et al., 2012; Burdisso et al., 2013). PTP1B dephosphorylates the autoinhibitory tyrosine of Src (Tyr 529 in mouse), contributing to its activation (Arregui et al., 1998; Bjorge et al., 2000). BiFC (bimolecular fluorescence complementation) analysis demonstrated that ER-bound PTP1B targets Src associated with the plasma membrane in contact with the substrate (Monteleone et al., 2012).

Src family kinases are transiently activated upon fibroblast adhesion to fibronectin (Kaplan et al., 1995; Zhang et al., 2008). Src- and integrin-dependent downregulation of RhoA activity occurs in a similar early temporal window after adhesion (Arthur et al., 2000; Danen et al., 2002). During this period, cells extend an F-actin rich lamellipodium and assemble small adhesions at the periphery (Alexandrova et al., 2008; Choi et al., 2008; Roca-Cusachs et al., 2013). Failure to downregulate RhoA induces abnormal development of prominent actin stress fibers and reduces cell spreading, likely as a consequence of increased acto-myosin-dependent contractility (Arthur and Burridge, 2001; Peacock et al., 2007).

Two major fibronectin receptors in fibroblasts, α5β1 and αvβ3, are able to transiently downregulate RhoA after cell attachment to fibronectin; however only α5β1 induces the later phase of RhoA activation (Danen et al., 2002). Differences among integrin signaling pathways may be partly related to their selective association and activation of downstream effectors. For example, β3 integrin, but not β1, interacts directly with and activates Src (Hruska et al., 1995; Chellaiah et al., 1996; Obergfell et al., 2002; Arias-Salgado et al., 2003; Courter et al., 2005). In fibrinogen-stimulated platelets, the association of PTP1B to the αIIbβ3 integrin/Src complex is required for Src activation, platelet spreading and clot retraction (Arias-Salgado et al., 2005).

In a recent study we showed that PTP1B null (KO) cells failed to downregulate RhoA activity and to induce Rac1 activity after attachment to fibronectin, as PTP1B-reconstituted (WT) cells did (Burdisso et al., 2013). We hypothesized that a consequence of PTP1B modulation of GTPase activities is the reduction of contractile forces at the cell periphery. In this work, we provide compelling evidence supporting this hypothesis, showing that PTP1B is required for efficient β3 integrin-dependent activation of Src/FAK signaling, which represses myosin activity and contractility. Our analysis further reveals that this process is crucial for lamellipodium and cell-matrix adhesion assembly during spreading.

## RESULTS

### PTP1B is required for early and transient integrin-dependent Src activation

**Fig. 1.**
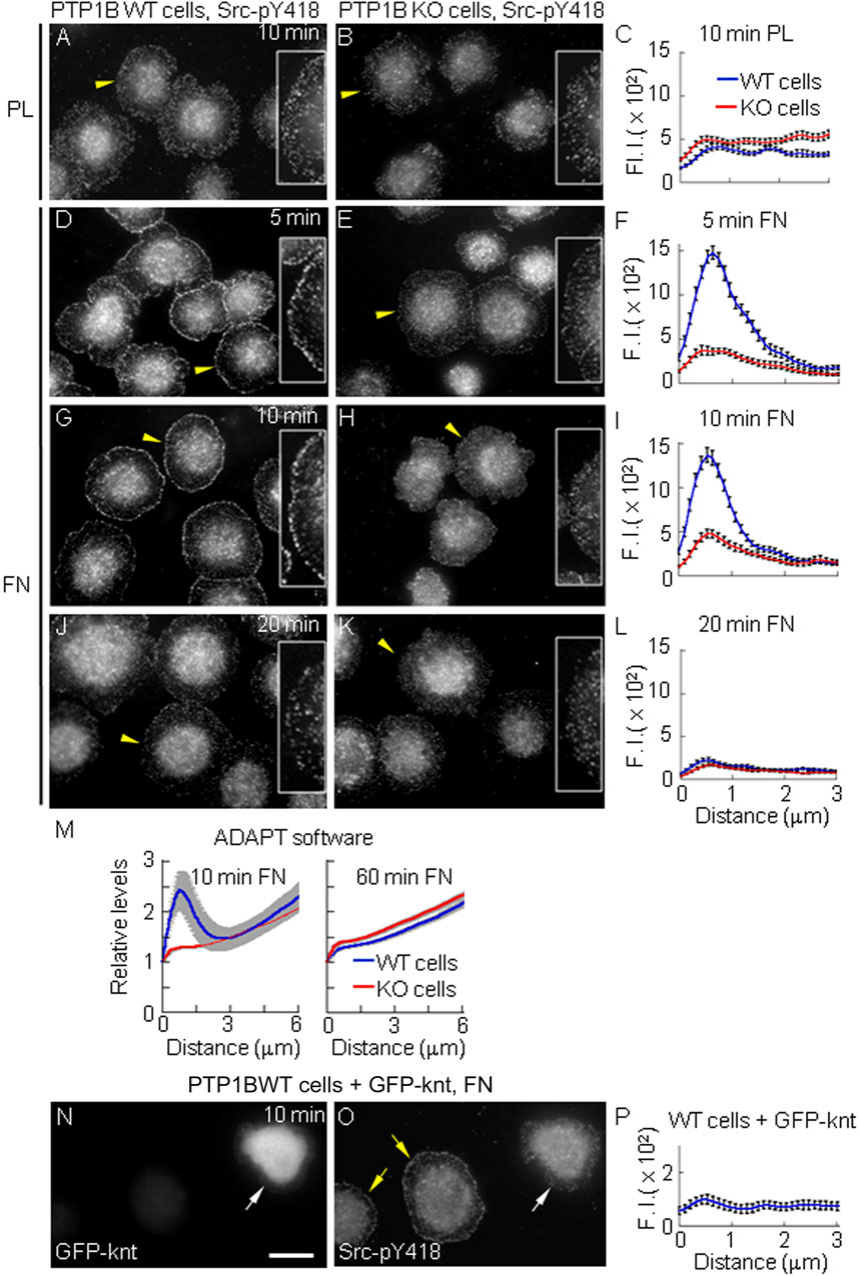
**Src is transiently activated at the cell periphery in a PTP1B- and integrin-dependent manner during early stages of spreading.** WT and KO cells plated on (A-C) polylysine or (D-O) fibronectin for different times, indicated in panels, and immunolabeled for Src-pY418. Yellow arrowheads indicate cells magnified at 200% in the insets, and showing the label distribution at peripheral regions. (C,F,I,L,P) Quantification of Src-pY418 along line scans orthogonal to the cell edge (0 µm). Values (in arbitrary units) represent mean±s.e.m. from >20 cells per condition. In F and I differences between mean values at the peak were statistically significant (*P*<0.0001, two-tailed Student's *t*-test). (M) Distribution of Src-pY418 signal from the cell border (distance=0 µm) to the cell center analyzed using the ADAPT software. Lines represent the mean of the Src-pY418 signal normalized to the first value at the cell border, and grey shading represents s.e.m. >45 cells analyzed per condition. (N-P) WT cells transfected with GFP-knt to induce the collapse of the peripheral ER. A representative field shows a transfected cell (white arrow) and two non transfected cells (yellow arrows). (P) The Src-pY418 signal no longer accumulates at the cell periphery in transfected cells (*n*=15). Scale bar: 20 µm.

Integrin stimulation induces Src and FAK activity (Burridge et al., 1992; Hanks et al., 1992; Lipfert et al., 1992; Kaplan et al., 1995). PTP1B is essential for αIIbβ3 activation of Src in platelets (Arias-Salgado et al., 2005). However, the spatiotemporal coordinates of these events are unknown. To address this issue we monitored active Src by immunodetection of Src phosphoTyr-418 in cell lines derived from PTP1B null mice, KO cells, and reconstituted with wild type PTP1B, WT cells (Haj et al., 2002). Serum-starved cells were plated for 5, 10, 20, 30 and 60 min on polylysine, as an unspecific substrate, and fibronectin, as a substrate to stimulate integrins. At all time points after plating on polylysine (only the 10 min time point is shown), Src-pY418 distributed throughout the cell, with no accumulation at the periphery in both, WT and KO cells (Fig. 1A,B). In contrast, at 5 and 10 min post-plating on fibronectin, Src pY418 was strongly accumulated at a peripheral ring of puncta in WT cells (Fig. 1D,G). This signal declined with time, being undetectable by 20 min post-plating (Fig. 1J). Src-pY418 did not accumulate at the periphery of KO cells plated on fibronectin at any time point (Fig. 1E,H,K). We quantified the peripheral signal by two methods, one measuring the fluorescence intensity along line scans orthogonal to the cell margin (Fig. 1C,F,I,L) (>20 cells per condition, cell border=0 µm). The magnitude of Src-pY418 fluorescent signal at the peripheral ring of puncta in WT cells did not correlate with cell area (not shown), and, on average, was 6-fold higher than the signal in the inner lamella (Fig. 1F,I, WT cells). We also used an automated method based on the ADAPT software (Barry et al., 2015) to quantify the Src-pY418 signal in cell contours obtained successively from the cell border to the cell center, with similar results (Fig. 1M). Antibody incompatibility precluded simultaneous examination of total Src and Src-pY418 in the same cells. Independent analysis using a pan-Src antibody did not reveal Src accumulation at the periphery of WT and KO cells (Fig. S1A-C). The specificity of Src-pY418 labeling was confirmed in SYF cells, which do not express Src, Fyn and Yes members of the Src family (Klinghoffer et al., 1999). SYF cells plated for 10 min on fibronectin show a faint perinuclear Src-pY418 signal (Fig. S1D,F). As expected, SYF cells expressing HA-tagged Src display a peripheral ring of Src-pY418 puncta, similar to that observed in WT cells. The total exogenous Src (revealed by HA labeling), did not accumulate at the cell periphery, as the endogenous pool (Fig. S1E,F). These results demonstrate that PTP1B and integrin stimulation are both required for transient Src activation at the periphery of spreading cells.

High resolution fluorescence microscopy reveals the localization of ER-bound PTP1B in the cell periphery, reaching the lamellipodium and the ring of active Src puncta (Fig. S1H-N). To examine whether peripheral ER positioning was required for Src activation, we transfected WT cells with a soluble GFP-kinectin construct (GFP-knt) which uncouples the ER from microtubules, leading to the collapse of the peripheral ER (Santama et al., 2004), and inhibition of cell spreading and migration (Zhang et al., 2010). GFP-knt impaired the peripheral distribution of ER-bound PTP1B without affecting the distribution of microtubules (not shown). At 10 min post-plating, transfected cells display similar area and adhesion patterns to non transfected cells (not shown). However, transfected cells lack the peripheral ring of Src-pY418 puncta (Fig. 1N-P), suggesting that peripheral positioning of ER-bound PTP1B promotes efficient integrin-dependent activation of Src.

### PTP1B cooperates with β3 integrin to activate Src in the lamellipodium

**Fig. 2.**
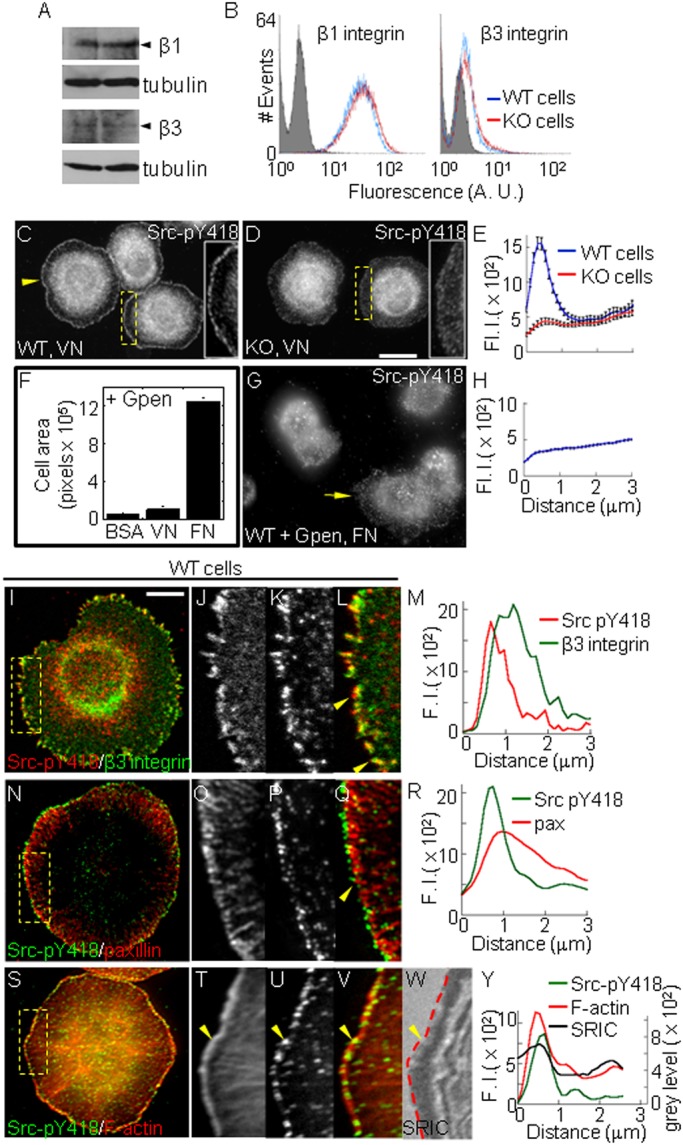
**PTP1B promotes β3 integrin-dependent aggregation and activation of Src at the lamellipodium.** (A)Western blot analysis of total β1 and β3 integrin expression in WT and KO cells. A representative result of two independent experiments is shown. (B) Flow cytometry analysis of surface β1 and β3 integrin expressions. Shaded area represents the signal of an isotype control antibody. Representative plots of two experiments run in duplicate are shown. (C,D) WT and KO cells plated on vitronectin for 10 min and labeled for Src-pY418 (insets are 200% magnifications of boxed regions). The yellow arrowhead points the peripheral ring of Src-pY418 puncta in WT cells. (E) Quantification of Src-pY418 label from line scans (*n*=20 cells, differences between mean values at the peak were statistically significant *P*<0.0001, two-tailed Student's *t*-test). (F) WT cells pre-incubated with GPen were plated on BSA-, vitronectin- or fibronectin-coated coverslips for 10 min. Adherent cells were labeled with phalloidin-TRITC and the total cell area was measured. Represented are mean±s.e.m. (G) WT cells were treated with GPen peptide, plated on fibronectin-coated coverslips and immunolabeled for Src-pY418. The yellow arrow points a spreading cell lacking the peripheral ring of Src-pY418. (H) Quantification of peripheral Src-pY418 signal (*n*=11 cells). Scale bar, 20 µm. (I-L) WT cells expressing β3 integrin-GFP plated on fibronectin and immunolabeled for Src-pY418. (J-L) Enlarged views show overlapping of Src-pY418 and β3 integrin-GFP at the cell periphery (yellow arrowheads). (M) Intensity profiles of β3 integrin-GFP (green line) and Src-pY418 (red line) signals along line scans traced over peripheral aggregates (0=distal pole). (N) TIRFM image of a representative cell double-labeled for Src-pY418 and paxillin. (O-Q) Enlarged views showing Src-pY418 localization at the distal poles of paxillin adhesions (yellow arrowhead). (R) Intensity profiles of Src-pY418 (green line) and paxillin (red line) signals (0=distal pole). (S-V) WT cells plated on fibronectin and labeled with phalloidin-TRITC, and Src-pY418. (T-V) Enlarged views show Src-pY418 and F-actin overlapping at the lamellipodium (yellow arrowhead). (W) SRIC image revealing the proximity of the membrane in contact with the substratum. The outer limit of the F-actin rich lamellipodium (shown in T) was overlaid (red dashed line). (Y) Intensity profiles of Src-pY418 (green line), F-actin (red line), and SRIC (black line) corresponding to line scans taken from the cell edge (0 µm) into the cell center. Scale bar, 10 µm. Values (in arbitrary units) in E,H represent mean±s.e.m. from >20 cells per condition.

Fibroblasts express two prominent fibronectin receptors, α5β1- and αvβ3-integrins; the latter is also a specific vitronectin receptor (Hynes et al., 1989; Bossy and Reichardt, 1990). Western blot and flow cytometry analyses reveal similar levels of total and surface expression of β1 and β3 integrin receptors in WT and KO cells (Fig. 2A,B).

To assess whether PTP1B and αvβ3 cooperate to activate Src, starved WT and KO cells were plated for 10 min on vitronectin and analyzed with anti-Src-pY418. A strong fluorescent signal accumulated in a ring of peripheral puncta only in WT cells (Fig. 2C-E). To determine whether αvβ3 function was also required for Src-pY418 activation on fibronectin, in which α5β1 heterodimer is functional, WT cells were incubated with the cyclic GpenGRGDSPCA (GPen) peptide. At the concentration used (1 mM), Gpen efficiently inhibits the vitronectin receptor but does not block cell attachment to fibronectin (Fig. 2F, Pierschbacher and Ruoslahti, 1987; Arregui et al., 1998). Cell spreading was reduced by GPen; however, in the fraction of spreading cells Src-pY418 accumulation at the cell periphery did not occur (Fig. 2G,H). Similar results were obtained in WT cells pre-incubated with a function-blocking anti-β3 integrin (not shown). Although we cannot rule out the contribution of additional vitronectin receptors, like αvβ1 and αvβ5 integrins, our results implicate that PTP1B and β3 integrin cooperate to activate Src at peripheral puncta.

To determine whether active Src puncta colocalize with adhesions, WT cells were transfected with β3 integrin-GFP and immunolabeled for Src-pY418. Confocal sections at the cell-substratum interface reveal that peripheral Src-pY418 puncta colocalize with the distal tips of β3 integrin aggregates (Fig. 2I-M). The distal localization of Src-pY418 in adhesive puncta was confirmed by TIRF microscopy (Fig. 2N-R). We determined whether the peripheral ring of adhesive puncta in WT cells localize within the lamellipodium, a thin layer of peripheral cytoplasm rich in actin filaments and accessory proteins like filamin, α-actinin and cortactin, among others (Small et al., 2002). Double immunofluorescence analysis revealed that Src-pY418 puncta overlapped with the peripheral labeling of F-actin, cortactin and α-actinin (Fig. 2S-V, only F-actin is shown). Surface reflectance interference contrast (SRIC) analysis reveals the membrane proximity to the substrate within the nanometer range, with low and high reflectance intensities representing membrane regions more or less close to the substrate, respectively (Weber, 2003; Monteleone et al., 2012). Src-pY418 puncta and lamellipodial markers juxtaposed to a low reflectance region at the cell margin (Fig. 2W,Y). Collectively, these results suggest that PTP1B promotes Src activation at new adhesions sites assembled within the lamellipodium.

### PTP1B promotes integrin-dependent FAK activation and paxillin phosphorylation

**Fig. 3.**
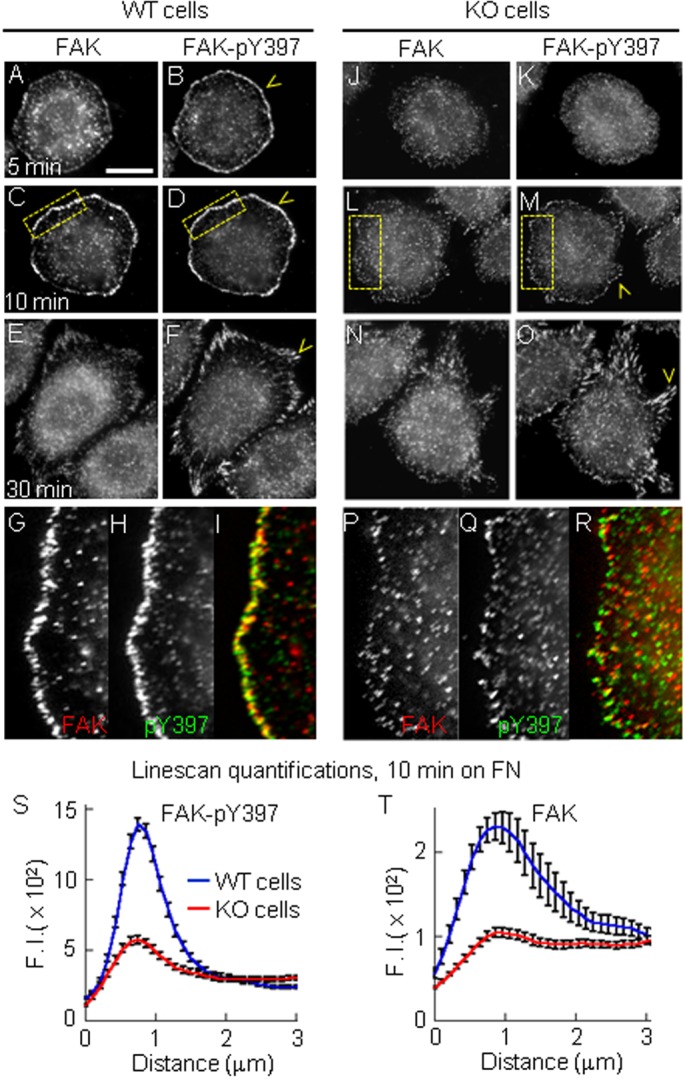
**PTP1B promotes early integrin-dependent FAK activation.** WT (A-I) and KO cells (J-R) plated on fibronectin for 5, 10 and 30 min. Cells were double-immunolabeled for FAK and FAK-pY397. (A-F) WT cells showing FAK-pY397 accumulation in a peripheral ring of puncta at 5 and 10 min, and in elongated peripheral adhesions at 30 min (yellow arrowheads). (G-I) Enlarged views (4×) of the boxed regions in C and D. (J-O) KO cells did not show FAK-pY397 accumulation at the cell periphery at 5 min post-plating, but localized in discrete peripheral puncta by 10 min (M) and in elongated peripheral adhesions by 30 min (O). (P-R) Enlarged views of the boxed regions in L and M. (S,T) Quantification of peripheral FAK-pY397 (S, *n*=42 cells) and FAK (T, *n*=32 cells) signal as described in Fig. 1. Differences between mean values at the peak were statistically significant *P*<0.0001, two-tailed Student's *t*-test. Scale bar, 20 µm. Values (in arbitrary units) in S,T represent mean±s.e.m. from >20 cells per condition.

Integrin aggregation induces autophosphorylation of FAK at tyrosine 397, creating a binding site for the Src-homology 2 domain of Src. Src phosphorylates and promotes the full activation of FAK (Schaller et al., 1994; Xing et al., 1994; Calalb et al., 1995; Mitra and Schlaepfer, 2006). The active Src/FAK complex phosphorylates the adaptor protein paxillin at Tyr-31 and Tyr-118 residues after cell-matrix adhesion (Schaller and Parsons, 1995; Bellis et al., 1997; Schaller et al., 1999). We examined the spatiotemporal activation of FAK in starved WT and KO cells plated on fibronectin, double labeled for FAK and FAK-pY397. FAK and FAK-pY397 accumulated in a peripheral ring of puncta at 5 and 10 min in WT cells (Fig. 3A-D,G-I). These puncta localized within the lamellipodium. FAK-pY397 signal persisted at high intensity in elongated peripheral adhesions at 30 and 60 min post-plating (Fig. 3E,F; only the 30 min time point is shown). KO cells did not show detectable FAK and FAK-pY397 accumulation at the cell periphery at 5 min post-plating (Fig. 3J,K), but discrete FAK-pY397 puncta were detected at the cell periphery by 10 min (Fig. 3M,Q,R) and the signal increased in elongated peripheral adhesions at 30 and 60 min post-plating (Fig. 3O, only the 30 min time point is shown). Quantification of the fluorescence intensity along line scans perpendicular to the cell border at 10 min post-plating revealed that the FAK-pY397 signal in WT cells was 3-fold higher than in KO cells (Fig. 3S). Likely, this result could be explained by deficient FAK aggregation at the periphery of KO cells (Fig. 3T).

**Fig. 4.**
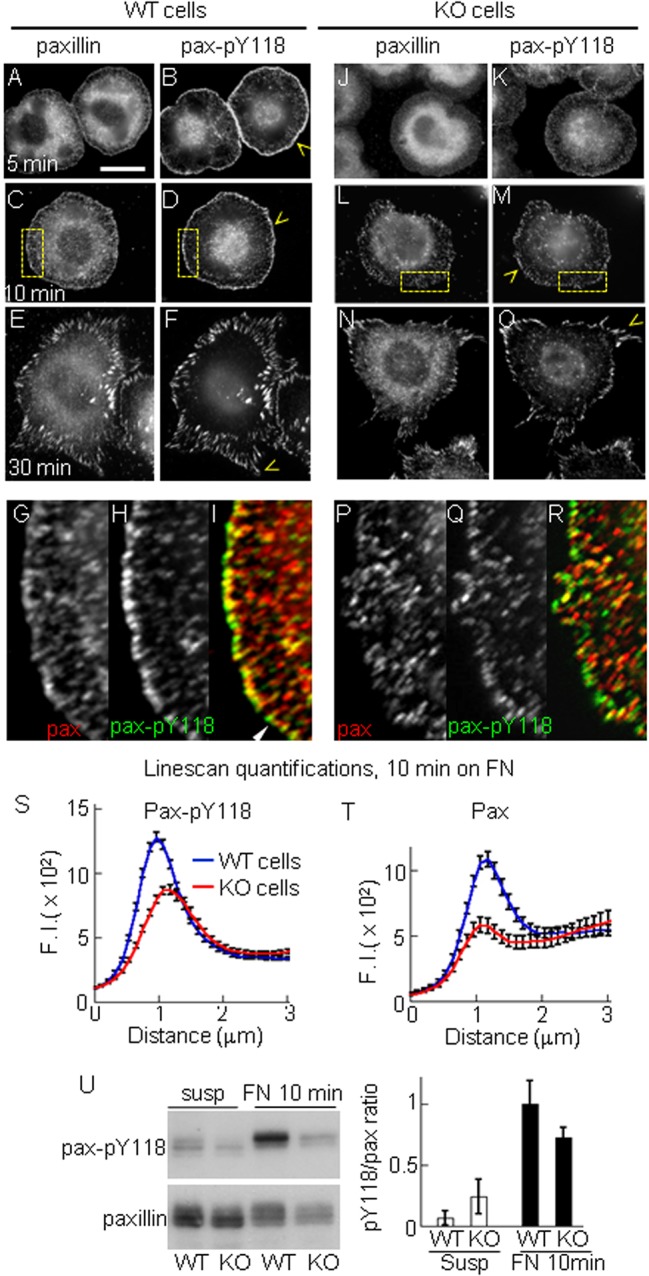
**PTP1B is required for integrin-dependent paxillin phosphorylation.** WT (A-I) and KO cells (J-R) plated on fibronectin for 5, 10 and 30 min after plating. Cells were double immunolabeled for paxillin and paxillin-pY118. (A-F) In WT cells, paxillin-pY118 strongly accumulated in a peripheral ring of puncta at 5 and 10 min and in elongated peripheral adhesions at 30 min (yellow arrowheads). (G-I) Enlarged views (4×) of the boxed regions in C and D. (J-O) In KO cells, paxillin-pY118 accumulation at the cell periphery was barely detectable at 5 and 10 min post-plating (K,M), but the signal increases at elongated peripheral adhesions by 30 min (O) (yellow arrowheads). (P-R) Enlarged views of the boxed regions shown in L and M. (S,T) Quantification of peripheral paxillin-pY118 (S, *n*=47 cells) and paxillin (T, *n*=29 cells) fluorescent signals as described in Fig. 1. Differences between mean values at the peak were statistically significant *P*<0.0001, two-tailed Student's *t*-test. (U) Western blot analysis of paxillin-pY118 and paxillin in cells kept in suspension or plated on fibronectin for 10 min. The plot represents averaged values of paxillin-pY118 normalized to total paxillin (*n*=3 experiments). Scale bar, 20 µm. Values (in arbitrary units) in S,T represent mean±s.e.m. from >20 cells per condition.

We next analyzed the phosphorylation of paxillin. In WT cells, paxillin and paxillin-pY118 accumulated at a peripheral ring of puncta at 5 and 10 min post-plating on fibronectin (Fig. 4A-D,G-I). In KO cells paxillin and paxillin-pY118 puncta were less developed than in WT cells (Fig. 4J-M,P-R). Quantification of paxillin-pY118 fluorescent signal at 10 min revealed that the peak intensity at the cell border was 40% lower in KO cells compared with WT cells (Fig. 4S). At later time points (30 and 60 min), WT and KO cells displayed similar elongated peripheral paxillin adhesions and strong pY118 signal (Fig. 4E,F,N,O; only the 30 min time point is shown). The lower phosphorylation of paxillin was confirmed by western blot analysis (Fig. 4U).

Compared to WT cells, KO cells also displayed reduced peripheral aggregation of β3 integrin and vinculin, lower phosphotyrosine content, and lower accumulation of lamellipodium markers like cortactin and F-actin (Fig. S2).

### PTP1B-dependent Src/FAK signaling represses myosin and promotes lamellipodium and adhesion assembly

**Fig. 5.**
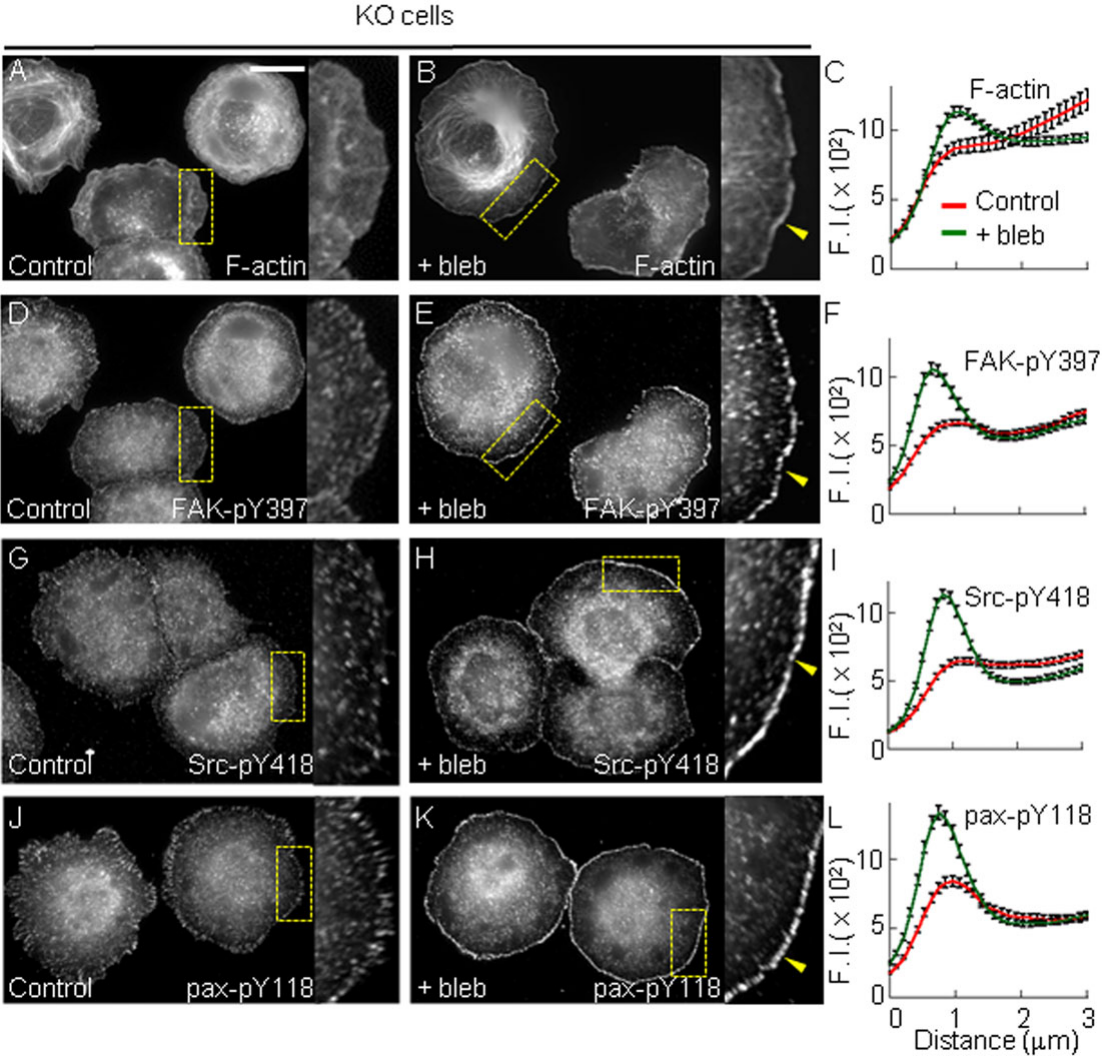
**Myosin inhibition restores lamellipodium and integrin-dependent signaling at the periphery of KO cells.** KO cells were incubated with blebbistatin (+bleb) or vehicle (control) and plated for 10 min on fibronectin. Control cells (A) or +bleb cells (B) were labeled with Phalloidin-TRITC. (C) Quantification of fluorescence in line scans (*n*=30 cells). Control cells (D) or +bleb cells (E) labeled with anti-FAK-pY397. (F) Quantification of FAK-pY397 fluorescence signal (*n*=40 cells). Control cells (G) or +bleb cells (H) labeled with anti-Src-pY418. (I) Quantification of Src-pY418 signal (*n*=74 cells). Control cells (J) or +bleb cells (K) labeled with anti-paxillin-pY118. (L) Quantification of paxillin-pY118 signal (*n*=40 cells). The robust effect of blebbistatin on the distribution of F-actin, FAK-pY397, Src-pY418 and paxillin-pY118 is better appreciated in enlarged views (3×) of peripheral regions (yellow boxes). In all cases differences between mean values at the peak were statistically significant *P*<0.0001, two-tailed Student's *t*-test. Scale bar, 20 µm. Values (in arbitrary units) in C,F,I,L represent mean±s.e.m. from >20 cells per condition.

RhoA activity is transiently downregulated immediately after integrin stimulation, event that requires functional Src/FAK (Arthur et al., 2000; Ren et al., 2000; Pirone et al., 2006; Schober et al., 2007; Bass et al., 2008; Tomar and Schlaepfer, 2009), and PTP1B (Burdisso et al., 2013). RhoA activity stimulates myosin-driven contractile forces (Geiger and Bershadsky, 2001; Zaidel-Bar et al., 2003). We hypothesized that inefficient activation of the Src/FAK signaling pathway in KO cells impairs lamellipodium and adhesion formation due to enhanced myosin activity. To test this we examined whether myosin inhibition by blebbistatin restores integrin-dependent signaling, lamellipodium and adhesion assembly at the periphery of KO cells plated for 10 min on fibronectin. Blebbistatin did not affect these processes in WT cells (Fig. S3). Remarkably, in KO cells blebbistatin induced the appearance of a well defined F-actin rich lamellipodium, which is not present in control cells (Fig. 5A-C, Fig. S2M-O). Blebbistatin also strongly induced the assembly of a peripheral ring of puncta containing FAK-pY397, Src-pY418 and paxillin-pY118 (Fig. 5D-L) as well as paxillin and β3 integrin (Fig. S4). Interestingly, blebbistatin did not have effect at longer times after plating (30 and 60 min, Fig. S3). Thus, attenuation of myosin activity in KO cells compensates for the lack of PTP1B function without altering the normal dynamics of peripheral puncta, which are undetectable by 30 min post-plating in both WT and KO cells. Unrestricted myosin-dependent mechanical strain at the cell periphery may promote Rac1 inhibition by FilGAP, a filamin A-associated Rac GAP that can suppress the formation of Rac-dependent lamellipodia (Shifrin et al., 2009; Ehrlicher et al., 2011; Nakamura, 2013). In agreement with this, expression of constitutive active Rac1 L61 in KO cells restored the lamellipodium formation (Fig. S4F-J), but it was unable to induce the assembly of peripheral adhesion puncta (not shown).

**Fig. 6.**
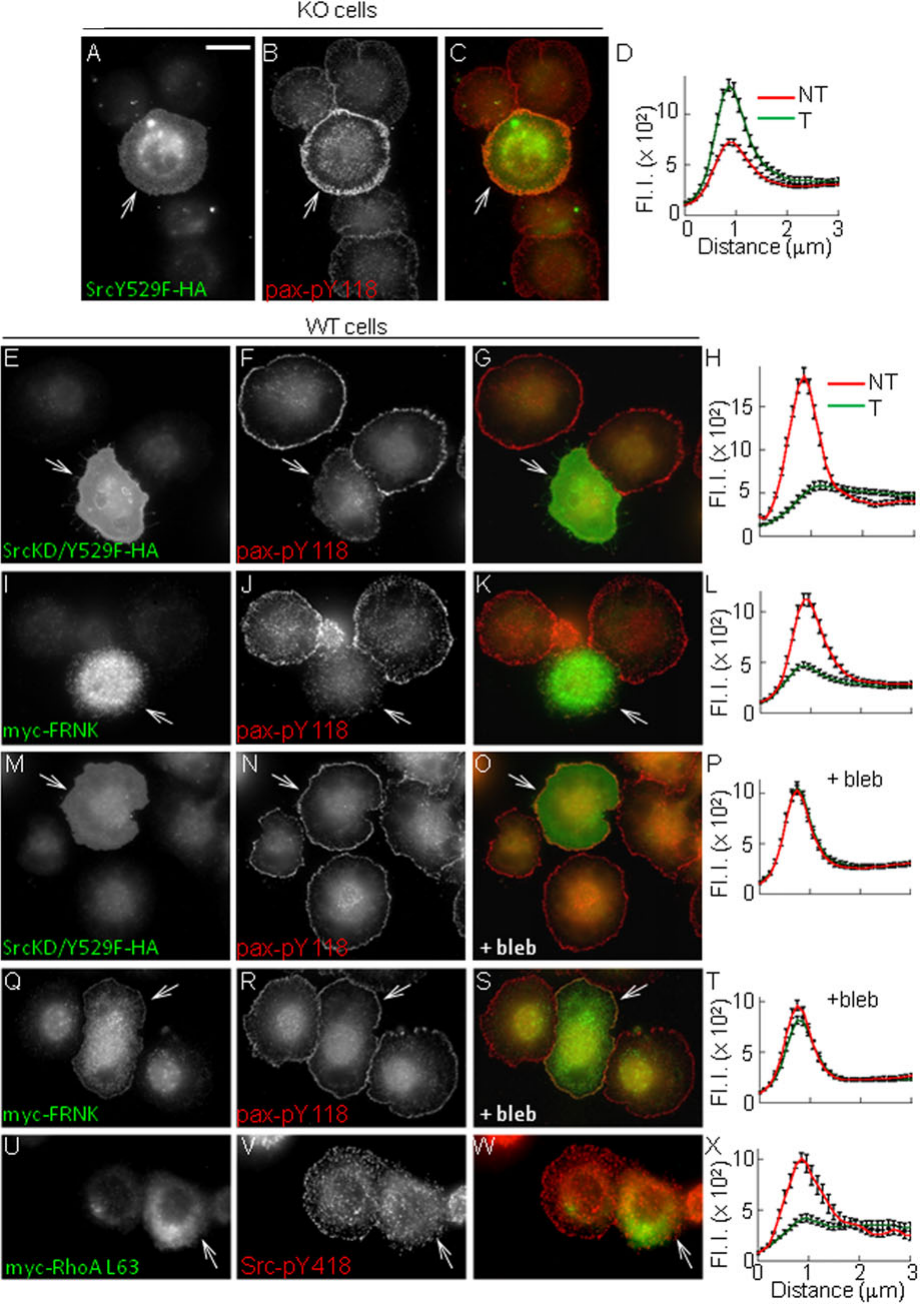
**Src-FAK-signaling and RhoA downregulation promote adhesion formation.** In all conditions cells were plated on fibronectin for 10 min. (A-C) KO cells expressing constitutively active SrcY529F-HA were immunolabeled for paxillin-pY118 and HA. Transfected cells restored paxillin-pY118 accumulation at peripheral puncta (white arrow). (D) Quantification of paxillin-pY118 signal as described in Fig. 1. Transfected (T=29 cells, green line) and non transfected (NT=64 cells, red line) cells. (E-L) WT cells expressing SrcKD/Y529F-HA mutant, to inhibit Src-dependent signaling (E-H), or myc-FRNK, to inhibit FAK function (I-L). Cells were immunolabeled for paxillin-pY118 (red signal) and HA/myc tags (green signals). Transfected WT cells (white arrows) show a significant reduction of paxillin-pY118 accumulation at cell margins compared to non transfected cells. (H,L) Quantification of paxillin-pY118 signal (T=30 cells, NT=23 cells). (M-T) WT cells expressing SrcKD/Y529F-HA (M-P) or myc-FRNK (Q-T) were incubated with blebbistatin and plated in the presence of the drug. Cells were immunolabeled for paxillin-pY118. Note that transfected cells (white arrows) display similar paxillin-pY118 accumulation at the periphery as non transfected cells. (P,T) Quantification of paxillin-pY118 signals (T=25 cells, NT=28 cells). (U-X) WT cells expressing active myc-RhoA L63 were immunolabeled for myc and Src-pY418. Transfected cells (white arrows) show significant reduction of Src-pY418 signal at the cell periphery. (X) Quantification of Src-pY418 signal (T=24 cells, NT=22 cells). (D,H,L,X) Differences between mean values at the peak were statistically significant *P*<0.0001, two-tailed Student's *t*-test. Scale bar, 20 µm. Values (in arbitrary units) in D,H,L,P,T,X represent mean±s.e.m. from >20 cells per condition.

Our results suggest that early after cell contact with the matrix, PTP1B cooperates with β3 integrins to activate a Src/FAK signaling pathway leading to the transient repression of RhoA and myosin-dependent contractility, allowing adhesion and lamellipodium assembly. To further substantiate this idea, we tested a number of predictions. First, artificially increasing Src/FAK signaling in KO cells should promote the formation of new adhesions at the cell periphery. To test this, we expressed a constitutive active Src mutant (Src Y529F) in KO cells. Transfected cells showed enhanced accumulation of paxillin-pY118 at a ring of peripheral puncta compared to non transfected KO cells (Fig. 6A-D). Second, impairing Src/FAK signaling in WT cells should reproduce the effects caused by PTP1B deficiency in KO cells. WT cells transfected with a dominant negative mutant of Src, SrcKD/Y529F (Mukhopadhyay et al., 1995; Burdisso et al., 2013) or with FRNK (FAK-related non kinase), a dominant negative mutant of FAK (Richardson and Parsons, 1996), displayed a significant reduction of Src-pY418 (not shown) and paxillin-pY118 at the cell periphery in comparison with non transfected WT cells (Fig. 6E-L). Third, inhibition of myosin should reverse the effect of Src/FAK impairment in WT cells. In fact, incubation of WT cells expressing SrcKD/Y529F or FRNK with blebbistatin completely reversed the negative effect of the constructs and restored Src-pY418 (not shown) and paxillin-pY118 puncta to levels similar to non transfected cells (Fig. 6M-T). Fourth, we predicted that expression of constitutively active RhoA in WT cells should impair adhesion formation and spreading. In fact, cells transfected with RhoA L63 have reduced spreading capacity (not shown), lack of paxillin-pY118 (not shown) and Src-pY418 accumulation at the cell periphery compared to non transfected cells (Fig. 6U-X).

### PTP1B decreases peripheral cell contractility during spreading

**Fig. 7.**
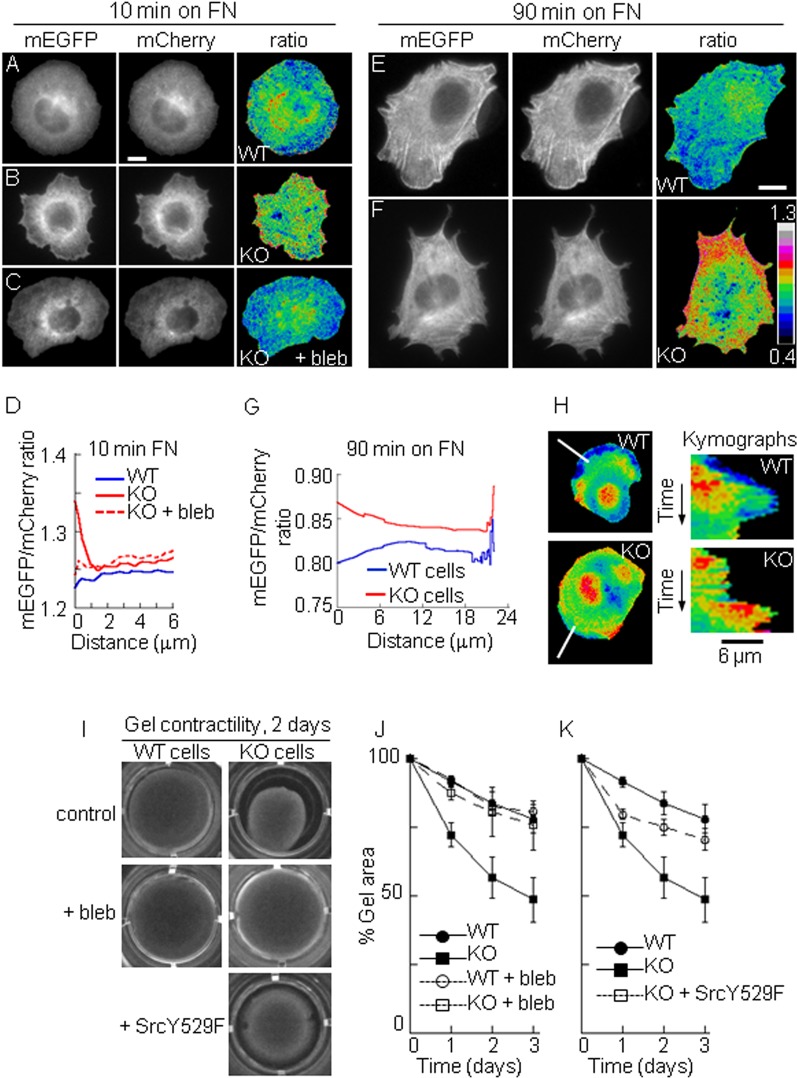
**PTP1B decreases cell contractility.** (A-G) Peripheral forces determined by the FLNA-CS biosensor in cells plated for 10 (A-D) and 90 (E-G) min and then fixed. WT (A,E), KO (B,F), and KO cells treated with blebbistatin (C) were processed to obtain mEGFP/mCherry ratio images. (D,G) Signal quantification along line scans (D) or using ADAPT software (G). Cell edge=0 µm, >20 cells analyzed per condition. (H) Sensor response in migrating cells. Representative kymographs showing the FLNA-CS signal variations along line scans (in white) normal to the leading edge of migrating cells (Movie 1). (I-K) Contractility of collagen gels containing WT or KO cells. (I) Representative paired images of gels after 2 days in culture. In (+bleb), blebbistatin was present since the beginning of the experiment. In (+SrcY529F), gels included KO cells transfected with constitutively active Src. (J,K) quantification of gel area over time. Each point represents the mean±s.e.m. of four experiments. Scale bar, 8 µm.

PTP1B regulates cell-matrix adhesion and motility through dephosphorylation of adaptors and scaffolds associated to integrin receptors, including p130Cas (Liu et al., 1996), paxillin (Takino et al., 2003), and α-actinin (Zhang et al., 2006; Burdisso et al., 2013). Our current data suggest an additional role of PTP1B promoting the transient suppression myosin-dependent contractility at the cell cortex during spreading. We evaluated the contractility at the cell cortex of WT and KO cells using a recently developed mechano-transduction sensor based on filamin A (Nakamura et al., 2014). Filamin A is an extended homodimeric protein that binds F-actin at the cell periphery (Nakamura et al., 2011; Razinia et al., 2012). The sensor, named FLNA-CS (Filamin A conformational sensor), consists of a FRET pair designed to quench the fluorescence of monomeric EGFP when the conformation of filamin is closed, as it occurs in the absence of cytoskeletal forces, and to unquench it (with fluorescence emission) when its conformation is open, as expected when contractile forces develop. The sensor has mCherry added at its C-terminal as an internal control to normalize for probe concentration. Determination of mEGFP/mCherry ratios in a pixel by pixel basis of cell images allows the reconstruction of two-dimensional maps of the sensor signal response. WT and KO cells expressing the FLNA-CS sensor were plated on fibronectin for 10, 60 and 90 min and analyzed in absence or presence of serum, with similar results. At 10 min after plating, WT cells showed low FLNA-CS signal at the cell periphery (Fig. 7A,D). By contrast, KO cells showed a strong sensor signal response at the cell edge, which decreases to reach a lower plateau ∼2 µm away of the cell border (Fig. 7B,D). Similar results were observed in cells plated for 60 and 90 min (Fig. 7E-G, only the 90 min time point is shown). As expected, incubation of KO cells with blebbistatin eliminated the strong sensor response at the cell periphery (Fig. 7C,D), and cells plated on polylysine showed a relatively uniform response of the sensor throughout the cell, with a slightly higher basal level in KO cells compared to WT cells (not shown). As in Fig. 1 we used two methods to quantify the variations of the peripheral signal from the cell border (=0 distance in the plots) to the cell center (Fig. 7D,G). To examine further the spatiotemporal FLNA-CS sensor response in migrating WT and KO cells we performed time lapse and kymograph analysis. WT cells consistently displayed a low sensor response at the protruding leading edge (Fig. 7H and Movie 1). In contrast, the protruding leading edge in KO cells showed a more variable sensor response, alternating bursts of moderate and high signal.

Fibroblasts are able to contract collagen gels in a myosin-dependent manner (Meshel et al., 2005). To determine the impact of PTP1B function in the capacity of cells to remodel 3D collagen gels, we measured the contraction of floating collagen gels containing WT and KO cells in complete culture medium. Determinations over time reveal that gels containing KO cells are consistent and significantly (*P*<0.05, two-tailed Student's t-test) more contracted than those containing WT cells (Fig. 7I,J). This result could not be attributed to differences in cell number (data not shown). Blebbistatin reverses the contractility capacity of KO cells to the level of WT cells. However, blebbistatin does not have effect on the contractile capacity of WT cells. To determine the role of Src activity, which is impaired in KO cells, we expressed a constitutive active Src mutant (Src Y529F) in KO cells. Under this condition, the contraction of gels was reduced at all time points (Fig. 7I,K). These results indicate that PTP1B contributes to reduce myosin-dependent contractility at the cell periphery, with detectable consequences to the attached extracellular matrix.

## DISCUSSION

Early integrin-dependent signal transduction events include the activation of the non receptor protein tyrosine kinases Src and FAK, which are responsible for most tyrosine phosphorylation activity occurring in adhesion complexes (Guan, 1997; Mitra and Schlaepfer, 2006; Huveneers and Danen, 2009). An important downstream consequence of these early molecular events is the modulation of Rho family GTPases, which control fundamental aspects of cell behavior, including cell spreading and migration (Ridley et al., 2003; Scales and Parsons, 2011; Spiering and Hodgson, 2011). The fibronectin receptors expressed in fibroblasts, α5β1 and αvβ3, display overlapping and distinctive effects on modulating RhoA activity (Morgan et al., 2009; Schiller and Fässler, 2013). Both heterodimers induce transient downregulation of RhoA activity immediately after cell adhesion; however, only α5β1 promotes the subsequent increment of RhoA activity required for focal adhesion maturation (Danen et al., 2002). They also drive divergent migratory behaviors; α5β1 promoting thin cell protrusions and random cell migration, and αvβ3 promoting the extension of broad lamellipodia and persistent migration (Danen et al., 2005). Our previous work suggests that the migratory behavior of KO cells was compatible with a α5β1-mediated pattern of migration, likely reflecting an impaired αvβ3 function (Hernández et al., 2006; Burdisso et al., 2013). Here we demonstrate that PTP1B promotes lamellipodium and adhesion formation at the protruding cell edge by ensuring the efficient β3 integrin-dependent activation of Src/FAK signaling. WT cells attached to fibronectin, which stimulates β1 and β3 integrins, or vitronectin, which selectively stimulates β3 integrin, develop lamellipodium and adhesive puncta eliciting Src/FAK activation and paxillin phosphorylation. This induction is inhibited by blocking β3 integrin function and occurs inefficiently in KO cells. NIH3T3 fibroblasts expressing a dominant-negative mutant of PTP1B (C215S) (Arregui et al., 1998) recreate some of the phenotypes described in KO cells (data not shown), suggesting that results are not biased in the PTP1B WT and KO cell lines. Our results agree with previous findings in fibrinogen-stimulated platelets, showing that PTP1B is recruited to a αIIbβ3/Src complex and is essential for Src activation (Arias-Salgado et al., 2005). Between 5-15 min after plating most WT cells exhibit a circumferential lamellipodium and a rounded shape, compatible with isotropic spreading (Dubin-Thaler et al., 2004). As time progresses, cells become increasingly asymmetric in shape and peripheral Src activation become spatially and temporally restricted to random and transient protrusions (Gulyani et al., 2011). This may explain the limitation of our quantification methods to detect significant Src-pY418 signal at longer times post-plating.

How impaired Src/FAK signaling in KO cells relates to the inefficient adhesion and lamellipodium assembly at the cell periphery? It is well established that after fibroblast attachment to fibronectin RhoA activity is modulated in a biphasic manner (Lawson and Burridge, 2014). A first phase of RhoA downregulation occurs during the first 30 min after integrin stimulation, which is then followed by a stimulatory phase (Ren et al., 1999; Arthur et al., 2000). RhoA downregulation correlates temporally with strong induction of Src and FAK activities (Hanks et al., 1992; Burridge et al., 1992, Lipfert et al., 1992; Kaplan et al., 1995; Klinghoffer et al., 1999), and is abolished by inhibition or genetic deletion of Src and FAK (Arthur et al., 2000; Ren et al., 2000; Pirone et al., 2006). It has been proposed that transient repression of RhoA is required to promote cell spreading and to antagonize cell contractility (Arthur and Burridge, 2001; Flevaris et al., 2007). We recently demonstrated that integrin-dependent downregulation of RhoA after fibronectin attachment is impaired in KO cells (Burdisso et al., 2013). Here we demonstrate that PTP1B activation of Src/FAK signaling downregulates RhoA-myosin activity, and as a consequence restricts contractility at the cell cortex, events that are functionally linked to the development of the lamellipodium and adhesions. First, we showed that inhibition of myosin activity in KO cells restored the lamellipodium and the rim of adhesive puncta, and signaling, in similar magnitude and spatiotemporal coordinates to that observed in WT cells. Second, constitutive activation of Src/FAK signaling (by SrcY529F expression) in KO cells also rescued lamellipodium and adhesion assembly. Conversely, impaired Src/FAK signaling (by dominant negative Src and FRNK expression) and enhanced RhoA activity (by RhoA L63 expression) in WT cells strongly inhibited the formation of peripheral adhesions, mimicking, to some extent, the KO cell phenotype. Remarkably, if myosin is simultaneously inhibited in these manipulated WT cells, peripheral adhesions reappear and are indistinguishable from those in control WT cells, underscoring the critical role of Src/FAK-dependent inhibition of myosin activity and contractility to allow the assembly of the cytoskeleton and adhesions at the protruding cell edge.

The acute and robust effect of blebbistatin in KO cells argues against major structural alterations that could prevent the assembly of adhesions and the lamellipodium in these cells. Instead, our results suggest a defect in Src/FAK signaling, which ultimately results in the inefficient assembly of the lamellipodium and adhesions. In agreement with this view, migrating KO cells display lamellar extensions and adhesion complexes of short duration compared to WT cells (Burdisso et al., 2013). The enhanced contractility of KO cells reported in the present work may explain, at least partly, these previous results. Enhanced cell contractility alters the conformation of filamin, with concomitant modulation of its interaction with partners (Ehrlicher et al., 2011; Razinia et al., 2012; Nakamura et al., 2011). Filamin is a large homodimeric protein, which interacts with F-actin to form orthogonal branches and provide network flexibility (Nakamura et al., 2007, 2011; Razinia et al., 2012). Using a filamin-based, force sensor in live cells, allowed to visualize specific spatiotemporal force patterns in WT cells, with lower sensor responses associated with the cell cortex in active protrusions. These patterns are altered in KO cells, which show high sensor responses at the cell cortex, blocked by blebbistatin incubation. Mechanical strain applied to F-actin/filamin networks reconstituted *in vitro* induced opposed effects on filamin binding to partners, with an increase of β-integrin binding and a decrease of FilGAP association (Ehrlicher et al., 2011). Enhanced filamin interaction with β-integrin inhibits integrin activation (Kiema et al., 2006; Das et al., 2011), effect that could explain, at least in part, the absence of adhesion puncta at the periphery of KO cells. FilGAP promotes GTP hydrolysis in Rac1, inhibiting its activity (Nakamura, 2013). Rac is required for lamellipodium and focal complex assembly, and is induced by integrin stimulation (Geiger and Bershadsky, 2001; Ridley et al., 2003; Huveneers and Danen, 2009; Lawson and Burridge, 2014). We recently showed that integrin-dependent Rac induction is impaired in KO cells (Burdisso et al., 2013). Our new findings revealing an enhanced contractility in KO cells suggest a negative regulation of Rac1 through the dissociation of FilGAP from filamin, and/or reducing the availability of Rac1 GEFs, such as β-Pix (Kuo et al., 2011; Kutys and Yamada, 2014). In fact, expression of constitutively active Rac1L61 in KO cells restored the formation of an F-actin-rich lamellipodium (Fig. S4). However, Rac1L61 was insufficient to induce the assembly of a rim of peripheral vinculin and paxillin pY118 puncta (results not shown), suggesting that additional signaling branches depending on Src/FAK activation by PTP1B are required for adhesion assembly and growth (Zaidel-Bar and Geiger, 2010; Robertson et al., 2015). One important hub of the phospho-adhesome network is the adaptor protein paxillin, which is tyrosine phosphorylated by Src/FAK in response to fibronectin adhesion (Burridge et al., 1992; Deakin and Turner, 2008; Robertson et al., 2015). Our results show reduced levels of paxillin phosphorylation at peripheral puncta in KO cells. It has been shown that expression of the phosphomimetic mutant of paxillin, Y31E/Y118E in fibroblasts, increases lamellipodial protrusions and focal complexes (Zaidel-Bar et al., 2007). Expression of paxillin-Y31E/Y118E in KO cells did not rescue lamellipodium and peripheral puncta (results not shown), arguing that the main constraint in KO cells is likely an enhanced myosin-dependent contractility at the cell periphery. The higher FLNA-CS response and collagen contraction capacity observed in KO cells, compared to WT cells, demonstrate the medium- and long-range effects of PTP1B deficiency.

**Fig. 8.**
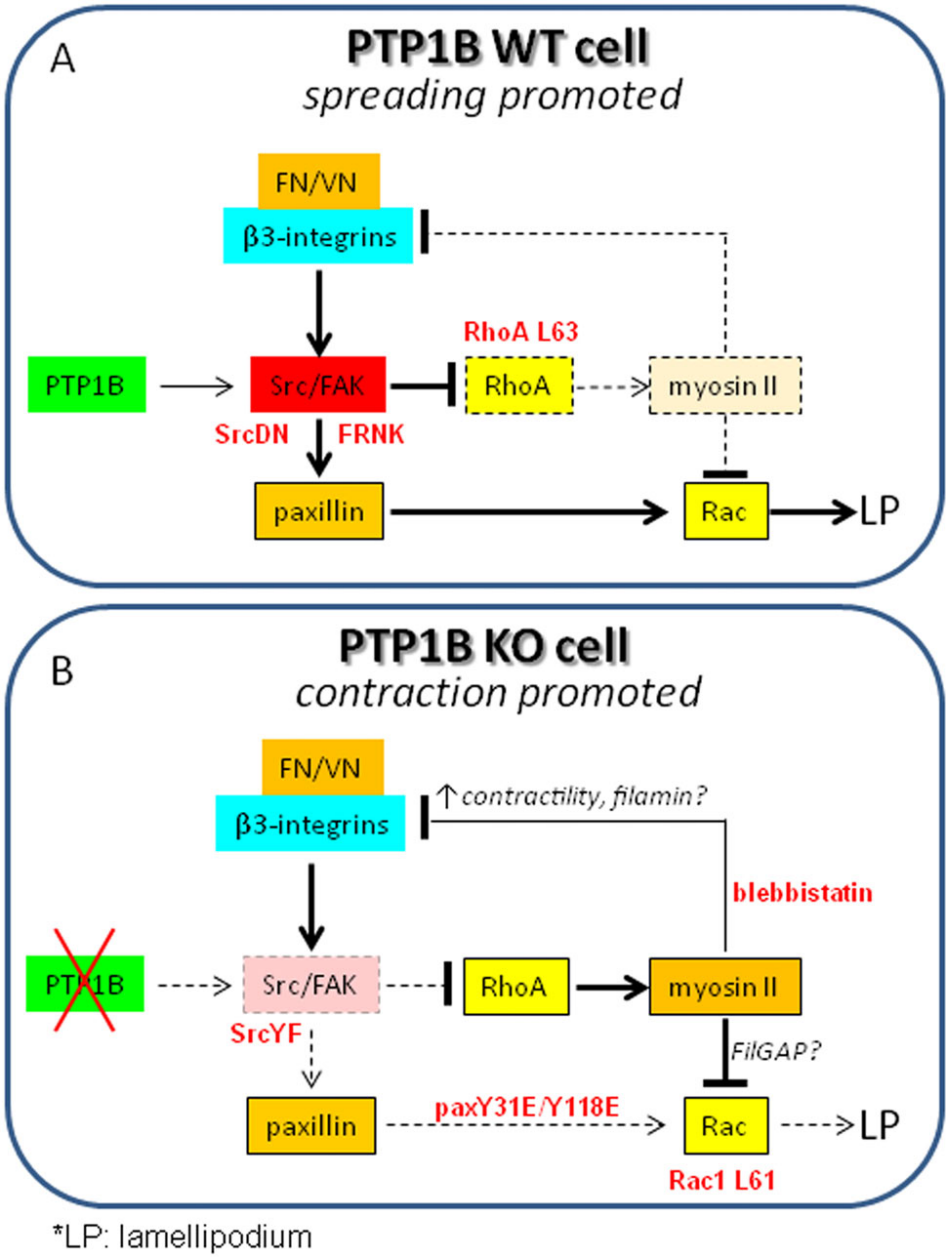
**PTP1B regulates cell contractility and spreading.** (A) In WT cells, PTP1B cooperate with β3 integrin to activate Src/FAK signaling and repress RhoA-myosin activation (dotted lines and boxes). These events modulate negatively acto-myosin contractility at the cell cortex, facilitating the assembly of a lamellipodium (LP) and peripheral adhesions required for cell spreading. Impairing Src/FAK signaling pathway by dominant negative Src (SrcDN) and FRNK expression, or increasing RhoA function by expression of RhoA L63, induces a KO cell phenotype. In WT cells, phosphorylation of paxillin promotes Rac1 activity. (B) In KO cells, early integrin-dependent Src/FAK activation is impaired (dotted box). RhoA/myosin-dependent contractility is enhanced (↑), and as a consequence, filamin and FilGAP availability likely undermine integrin function and lamellipodium assembly, respectively. This condition facilitates cell contraction. Expression of constitutive active Src (SrcYF), or incubation with blebbistatin, restores peripheral adhesions and the lamellipodium. In KO cells Rac1 activity is not induced efficiently, likely by reduced phosphorylation of paxillin and other adaptors, and by negative regulation imposed by enhanced RhoA/myosin signaling. Expression of constitutively active Rac1 L61 rescues lamellipodium formation. Expression of the phosphomimetic paxillin Y31E/Y118E is not enough for adhesion assembly.

Our results support a model in which PTP1B cooperates with β3 integrin to set in motion a feed-forward mechanism at the cell periphery during initial stages of contact with the substratum. This mechanism involves activation of the Src/FAK signaling pathway and inhibition of RhoA-myosin activity. The biological consequence is a reduction of contractile forces at the periphery, generating permissive conditions for adhesion, lamellipodium assembly, and spreading (Fig. 8). Myosin deregulation in KO cells may have a wide range of physiological implications. Remarkably, we demonstrated a significant effect on collagen contraction. Higher contractile capacity of PTP1B-deficient cells may explain defects in clot retraction in platelets (Arias-Salgado et al., 2005), cell migration in fibroblasts (Hernández et al., 2006; Burdisso et al., 2013), axon elongation (Fuentes and Arrequi, 2009), and dendritic spine maturation (Fuentes et al., 2012).

## MATERIALS AND METHODS

### Cell culture and treatments

PTP1B null (KO) cells and PTP1B reconstituted (WT) cells (Haj et al., 2002) and SYF cells (ATCC) were cultured in high glucose DMEM containing L-glutamine, supplemented with 10% fetal bovine serum, penicillin and streptomycin (Invitrogen). Unless indicated, cells were serum-starved for 4 h, and then resuspended with 0.05% trypsin in PBS (137 mM NaCl, 2.7 mM KCl, 10 mM Na_2_HPO_4_, 1.8 mM KH_2_PO_4_, pH 7.4) containing 1 mM EDTA. Trypsin was neutralized with soybean trypsin inhibitor (Sigma-Aldrich). Cells (1×10^5^) were plated in plain DMEM on coverslips coated with poly-L-lysine, (150 μg/ml), fibronectin (20 μg/ml), both from Sigma-Aldrich, or with vitronectin (5 μg/ml) obtained from BD Biosciences. After 5, 10, 20, 30 and 60 min cells were fixed for subsequent analysis. When indicated, cells were pre-incubated with blebbistatin (20 µM, Sigma-Aldrich) for 60 min at 37°C, with G-Pen peptide (1 mM, GRGDSPCA, American Peptide Company), with anti-β3 antibody, or isotype control IgG (20 µg/ml), for 30 min at 4°C, before plating in the presence of the reagents. The effect on attachment and spreading was evaluated by microscopy using Metamorph (Molecular Devices).

### Antibodies and other labeling reagents

Phalloidin-TRITC, and monoclonal antibodies against vinculin (hVIN-1), HA (HA-7), c-myc (9E10) and α-tubulin (DM 1A) were from Sigma-Aldrich. Hamster anti-mouse CD29 (HMβ1-1), hamster anti-mouse CD61 (2C9.G2), hamster IgG isotype control and FITC goat anti-hamster IgG were from Biolegend, and HRP-conjugated rabbit anti-hamster IgG was from Abcam. Polyclonal antibody against FAK-pY397 was from Biosource International. Polyclonal antibodies against Src-pan, Src-pY418 and paxillin-p-Y118 were from Invitrogen. Monoclonal anti-paxillin (349), anti-phosphotyrosine (PY20), anti-PTP1B (15/PTP1B) and anti-FAK (77) were from BD Transduction Laboratories. Alexa Fluor 488- and Alexa Fluor 568-conjugated secondary antibodies were from Invitrogen. HRP-conjugated antibodies were from Jackson Immunoresearch.

### DNA constructs and transfections

Cells were transfected using Lipofectamine 2000 (Invitrogen) and processed 24 h post-transfection. The pCMV-myc-FRNK was provided by J. T. Parsons (University of Virginia); EGFP-knt by H. Yu (National University of Singapore); RhoA L63 and Rac1 L61 in pRK5-myc by A. Hall (Memorial Sloan-Kettering Cancer Center); and pSP73-β3-integrin by A. Teitelbaum (University of Washington). To obtain β3 integrin-GFP, β3 integrin cDNA was amplified by PCR and inserted into Bgl II/Age I sites of pEGFP-N1. Both proteins are joined by a GPVAT spacer. Transfected β3-GFP localizes in cell-matrix adhesions only when cells are seeded on fibronectin and vitronectin but not on laminin (not shown). GFP-PTP1B, mRFP-PTP1B D181A, Src-HA, SrcY529F-HA and Src KD/Y529F-HA were previously described (Hernández et al., 2006; Burdisso et al., 2013).

### Flow cytometry

Surface expression of β1 and β3 integrins in WT and KO cells was analyzed by flow cytometry. Cells (1×10^6^) were harvested with trypsin/EDTA, diluted with complete medium and centrifuged. Cells were resuspended in PBS/BSA 0.1%, and incubated on ice for 60 min with hamster anti-CD61, hamster anti-CD29, or hamster IgG isotype control (20 µg/ml). After washing with PBS, cells were incubated with FITC-conjugated goat anti-hamster (1:100) on ice for 60 min. Cells were washed, fixed in 1% paraformaldehyde, and analyzed on a FlowMax cytometer PASIII (Partec). Data were plotted using WinMdi 2.9 software (Bio-Soft Net).

### Microscope analysis

Cells were fixed with 4% paraformaldehyde in PBS (20 min), permeabilized with 0.5% Triton X-100 (5 min) and blocked with 3% BSA (1 h). Primary and secondary antibodies were incubated in a humid chamber for 1 h. Samples were mounted in Vectashield (Vector Laboratories) and observed through a 60×/1.4 NA objective in an Olympus FV1000 confocal microscope, or by wide-field in a Nikon TE2000-U microscope coupled to an ORCA-ER CCD camera (Hamamatsu). For SRIC, a cube with a green excitation filter, a UV dicroic mirror and without barrier filter was set in place in the epi-filter rotating turret. For TIRFM, cells were observed with a 60×1.45 NA objective in a Nikon TE2000-E inverted microscope coupled to an ORCA II ER CCD camera controlled by Metamorph. A 100 W mercury lamp was used for SRIC and wide-field observation, and a 488 nm argon laser and a 543 nm helium/neon laser for confocal and TIRFM. Penetration depth of the evanescent field (∼210 nm) was calculated as described (Monteleone et al., 2012).

Mechanical strain was determined using the FLNA-CS force sensor (Nakamura et al., 2014). Transfected WT and KO cells were fixed and analyzed by wide-field fluorescence microscopy. Incident light from a 100 W mercury lamp was attenuated to 25% using neutral density filters. Monomeric EGFP was imaged using a B-2E/C filter cube [excitation filter, 480/30, dicroic mirror 505 (LP), emission filter 535/40], and mCherry was imaged using filters in wheels (excitation filter 565/25, emission filter 620/60) in combination with a 86007bs dichroic mirror (Chroma). Images were acquired using binning 2 and exposure times ranging 500-1500 ms. For time lapse imaging, serum-starved cells were plated on MatTek's coverglass-bottom 35 mm culture dishes coated with fibronectin in 2.5 ml phenol red-free DMEM, supplemented with 25 mM HEPES, 0.8 U/ml OxyFluor (Oxyrase), and 10% fetal bovine serum. Dishes were placed on the microscope stage enclosed within an incubator system set at 37°C (Solent Scientific). Cells were imaged 60 min after plating through a 60×1.4 NA objective. Incident light was attenuated to 3% using neutral density filters, camera binning was set to 4, and exposure times were 500-900 ms. Light between acquisitions (every 1 min) was shuttered (Sutter Instrument). All peripherals were controlled with Metamorph. Image stacks were built and processed using ImageJ (NIH).

### Western blots

WT and KO cell suspensions or attached on fibronectin-coated dishes (10 µg/ml), (1×10^6^ cells per condition) were lysed on ice with TBS (20 mM Tris-HCl pH 7.4, 137 mM NaCl) containing 1% Triton X-100, 2.5 mM NaVO_3_, 10 mM NaF and protease inhibitors (Sigma-Aldrich). Cell lysates were centrifuged at 13,600 ×***g*** for 15 min at 4°C and ∼30 µg of the supernatants were fractionated by SDS-PAGE and transferred to polyvinyl difluoride membranes. After blocking with 3% BSA, membranes were probed with primary antibodies (2 µg/ml) followed by peroxidase-conjugated secondary antibodies and revealed by ECL (SuperSignal West Femto Maximum Sensitivity Substrate, Thermo Scientific).

### Image analysis and quantitative procedures

All quantitative procedures were performed using ImageJ. Microscopy 12-bit images were corrected for shading and background-subtracted. The cell border was defined by thresholding. Variations of signal intensity at the cell periphery were quantified along four equidistant line scans (5 µm width and 3-6 µm length) orthogonal to the cell border, which in total covered 15-25% of the entire cell perimeter. Pixel values from the four line scans were averaged per cell. Means and s.e.m. values of more than 15 cells per condition were used for representation using Kaleidagraph (Synergy Software). As an alternative quantification method, we used the ‘ADAPT’ software (Barry et al., 2015) to calculate the mean fluorescence intensity of segmented image boundaries taken successively from the cell border to the cell center. The FLNA-CS sensor response was quantified according to published procedures (Nakamura et al., 2014), mEGFP and mCherry images were aligned (MultistackReg plugin), and the time lapse stacks corrected for bleaching (corr_blech050405, EMBL) before ratio calculation. For visualization, ratio images were pseudo-colored using the ratio lookup table. Kymographs of a representative time lapse experiment (Movie 1) were generated using the MultipleKymograph plugin for ImageJ. The ratio values were represented along line scans (1-pixel wide) orthogonal to the cell border in images recorded every min during 25 min.

### Collagen gel contraction assays

Type I collagen was prepared from rat tail tendons (Price, 1975). Gel contraction assays were performed as previously described (Jean et al., 2013). Briefly, collagen diluted in DMEM was adjusted to a pH 7.4 with 1 M NaOH and mixed with a cell suspension, so that 500 µl containing 0.6 mg collagen and 5×10^5^ cells was added to each well of 24-well cell culture plates (Costar), previously blocked with 3% BSA. Collagen was allowed to polymerize for 1 h at 37°C. Then, complete DMEM was added to the wells and collagen gels were gently detached using a pipette tip. Floating gels were incubated for 72 h at 37°C, and photographed every 24 h. Gel area was measured using ImageJ. When indicated, collagen gels were incubated with blebbistatin (20 µM) from the beginning of the experiment.
